# Development and Evaluation of a Natural Language Processing System for Curating a Trans-Thoracic Echocardiogram (TTE) Database

**DOI:** 10.3390/bioengineering10111307

**Published:** 2023-11-10

**Authors:** Tim Dong, Nicholas Sunderland, Angus Nightingale, Daniel P. Fudulu, Jeremy Chan, Ben Zhai, Alberto Freitas, Massimo Caputo, Arnaldo Dimagli, Stuart Mires, Mike Wyatt, Umberto Benedetto, Gianni D. Angelini

**Affiliations:** 1 Bristol Heart Institute, Translational Health Sciences, University of Bristol, Bristol BS2 8HW, UKangus.nightingale@bristol.ac.uk (A.N.); jeremy.chan@bristol.ac.uk (J.C.); m.caputo@bristol.ac.uk (M.C.); arnaldo.dimagli@bristol.ac.uk (A.D.); umberto.benedetto@bristol.ac.uk (U.B.); g.d.angelini@bristol.ac.uk (G.D.A.); 2 School of Computing Science, Northumbria University, Newcastle upon Tyne NE1 8ST, UK; 3 Faculty of Medicine, University of Porto, 4100 Porto, Portugal; alberto@med.up.pt; 4 University Hospitals Bristol and Weston, Marlborough St, Bristol BS1 3NU, UK; mike.wyatt@uhbw.nhs.uk

**Keywords:** electronic health records (EHR), Big Data, unstructured data, echo report, echocardiography analysis, natural language processing (NLP), data extraction, validation

## Abstract

Background: Although electronic health records (EHR) provide useful insights into disease patterns and patient treatment optimisation, their reliance on unstructured data presents a difficulty. Echocardiography reports, which provide extensive pathology information for cardiovascular patients, are particularly challenging to extract and analyse, because of their narrative structure. Although natural language processing (NLP) has been utilised successfully in a variety of medical fields, it is not commonly used in echocardiography analysis. Objectives: To develop an NLP-based approach for extracting and categorising data from echocardiography reports by accurately converting continuous (e.g., LVOT VTI, AV VTI and TR Vmax) and discrete (e.g., regurgitation severity) outcomes in a semi-structured narrative format into a structured and categorised format, allowing for future research or clinical use. Methods: 135,062 Trans-Thoracic Echocardiogram (TTE) reports were derived from 146967 baseline echocardiogram reports and split into three cohorts: Training and Validation (n = 1075), Test Dataset (n = 98) and Application Dataset (n = 133,889). The NLP system was developed and was iteratively refined using medical expert knowledge. The system was used to curate a moderate-fidelity database from extractions of 133,889 reports. A hold-out validation set of 98 reports was blindly annotated and extracted by two clinicians for comparison with the NLP extraction. Agreement, discrimination, accuracy and calibration of outcome measure extractions were evaluated. Results: Continuous outcomes including LVOT VTI, AV VTI and TR Vmax exhibited perfect inter-rater reliability using intra-class correlation scores (ICC = 1.00, *p* < 0.05) alongside high R^2^ values, demonstrating an ideal alignment between the NLP system and clinicians. A good level (ICC = 0.75–0.9, *p* < 0.05) of inter-rater reliability was observed for outcomes such as LVOT Diam, Lateral MAPSE, Peak E Velocity, Lateral E’ Velocity, PV Vmax, Sinuses of Valsalva and Ascending Aorta diameters. Furthermore, the accuracy rate for discrete outcome measures was 91.38% in the confusion matrix analysis, indicating effective performance. Conclusions: The NLP-based technique yielded good results when it came to extracting and categorising data from echocardiography reports. The system demonstrated a high degree of agreement and concordance with clinician extractions. This study contributes to the effective use of semi-structured data by providing a useful tool for converting semi-structured text to a structured echo report that can be used for data management. Additional validation and implementation in healthcare settings can improve data availability and support research and clinical decision-making.

## 1. Introduction

Electronic health records (EHR) have become increasingly important as they generate more and more data leading to “Big Data”, holding key insights into disease patterns and opportunities to optimise patient treatment. However, the reliance on semi-structured data is a major barrier in using EHR. These types of data do not have a fixed structure but have some type of organisation, generally through the use of tags, labels or metadata. While semi-structured data enables flexibility in data representation, they are limited in terms of the lack of standardisation, irregular organisation, ambiguity and interpretation issues. Extracting useful information and integrating semi-structured data can be difficult, necessitating the use of advanced techniques such as natural language processing and data mapping. When interacting with patients, healthcare workers frequently use freeform notes to record vital details [1].

The echo report based on echo imaging is the fundamental record of evidence for the diagnosis of a cardiovascular patient. The structure (e.g., valves, cardiac chambers and blood vessels dimensions) and functionalities (e.g., ejection fraction, global longitudinal strain) of the heart are examined and described in the echo report by the echocardiographer with a mix of semi-structured numerical data and free text descriptions. However, the extraction and generation of research data from the original document are challenging, mainly due to the narrative nature of the echo report, different reporting styles of echocardiographers, differences in echo devices (reporting platforms and vendor software) and hospital specific protocol differences. As such, the extraction of structured data from semi-structured echo reports, especially for statistical analyses, tends to be excessively time consuming and requires tremendous effort and cost, owing to its presentation as a narrative document.

While NLP models and tools were used to process data from psychiatry, X-ray radiography [2] and pathology [3] to distinguish healthy from diseased patients [4,5], they are not frequently used in echocardiography analysis.

Whilst the retrieval of categorised data from clinical data repositories is relatively simple, not all data are available in this format. Large organisations, such as the NHS, typically store large amounts of information in the form of free text, within which specific categories of data (e.g., aortic or mitral valve haemodynamics) cannot be easily retrieved for analysis. To enable the data contained within free text to be used for statistical analyses, it is necessary to both extract and convert that information into a structured format. However, there are significant challenges to the extraction process, which can be summarised as: (a) variability of language, which is often ambiguous and complex; (b) lack of standardisation; and (c) incomplete information and privacy concerns. This paper presents an approach that employs a natural language processing (NLP) system to handle variations in echocardiographic data to construct a comprehensive data repository by accurately categorising, extracting and organising semi-structured echocardiographic data.

The primary aim of this study is to improve data management and analysis for more effective use of echocardiographic data in research and clinical applications. To achieve this aim, we have defined two key objectives. Our primary objective is to create a structured moderate-fidelity echocardiogram database using automated data extraction and curation technologies. We envision this database being easily integrated with high-fidelity datasets such as EHRs [6], supporting the aim of effective echocardiographic data usage in research by promoting multimodal learning methodologies [7]. In parallel, our second objective is to meticulously identify factors that consistently display superior extraction performance. This aspect of research is critical in terms of future clinical applications, such as patient monitoring and the development of predictive risk scores, both of which have the potential to alter patient care and medical research.

We validated the proposed method by comparison of the NLP extraction with clinician’s annotations on a sample of the most common type of echocardiography examination, Trans-Thoracic Echocardiogram (TTE). Finally, the approach is scaled to extract all available echocardiogram free text reports within the local hospital.

The main contributions of the study are summarised as follows:

- Improved Data Extraction Using NLP. This study introduces and illustrates the usage of a GATE-based NLP system to extract comprehensive information from echocardiography reports. This approach substantially improves the efficiency while maintaining accuracy to extract meaningful insights from these reports by automating the data extraction process.
- Data-Driven Clinical Decision-Making. By utilising Big Data resources, this work contributes to the realisation of learning health systems. It provides the foundation for using data-driven [8] insights to help healthcare practitioners make precise diagnoses, identify at-risk patients, develop personalised treatment regimens and enhance medical research in complex patient populations. It enables healthcare workers to immediately identify critical parameters and red flags in preoperative and postoperative settings, such as changes in aortic diameters or valve haemodynamics. This feature can also help to expedite surgical prioritisation, patient monitoring and waiting list administration.
- Opportunities for Research and Audits. The NLP system’s capabilities go beyond treating specific patients. It paves the way to advanced audit and research projects in cardiac care. With the use of this technology, researchers can monitor patterns and examine large datasets, advancing our knowledge of heart conditions and therapeutic outcomes.
- Interpretable Integration of Data. The study addresses the challenge of integrating both continuous and discrete data, such as qualitative ratings and quantitative measurements, in echocardiogram reports. The structured database generated by the NLP system offers a clear and interpretable representation of this combined information, facilitating its practical use.
- Potential for Broader Healthcare Applications. While the study focuses on echocardiography reports, the concepts and methods explored here can potentially extend to other investigative modalities, including CT scans. The NLP system’s ability to integrate with risk modelling approaches further expands its potential impact on healthcare research and practice.

In the next section we report on some related work.

## 2. Related Work

In [9], NLP algorithms were created and verified to identify aortic stenosis (AS) cases from echocardiography reports and compare their precision to diagnosis codes. The promise of NLP for enhancing case identification in population health was demonstrated by the NLP algorithms’ greater accuracy in detecting AS cases than diagnostic codes. An approach for obtaining numerical test results and associated descriptions from free-text echocardiogram reports was proposed in another study [10]. The system efficiently handled typos, synonyms and abbreviations by using corpus-independent algorithms and fuzzy matching to detect and pair expressions with measurement results. It was useful for processing large numbers of echocardiographic findings and showed potential for assisting medical research or clinical trial verification. In a different study, a method for turning heterogeneous echocardiographic medical notes into structured data based on NLP was provided [11]. The researchers developed a unique NLP-based extraction and processing programme, EchoInfer, to automate the extraction and organisation of the 80 frequently evaluated echocardiogram data items. By converting unstructured, semi-structured and structured data from echocardiograms into a format compatible with traditional analytical techniques, EchoInfer’s effectiveness and consistency were established. Another study evaluated the generalisability of Left Ventricular Ejection Fraction (LVEF) extraction modules, using a data set known as the TUCP EF year 3 corpus (including echocardiography and radiology data reports) [12]. The features for detecting LVEF information were examined, and NLP techniques based on a machine learning (ML) sequential tagger were deployed. The work made contributions by assessing how well previous LVEF extraction modules performed on the new corpus, and by examining how the amount of training data influenced the accuracy of the new NLP modules. Another study showed that NLP may be used to categorise stress echocardiography (SE) data and extracted variables that were frequently utilised in stress testing score models [13]. By synthesising data elements from the reports, the NLP system produced a valid summary and demonstrated high accuracy in criteria validity when compared to the reference standard. Construct validity was also evaluated [13], and the results demonstrated that, in line with prior findings, NLP-derived SE results effectively distinguished patients at short-term cardiac risk.

Transformer based models, such as the Bidirectional Encoder Representations from Transformers (BERT), have also been applied for extract knowledge from unstructured notes in the healthcare domain. Studies typically begin with standardisation of text by using the NLTK toolkit for case conversion, removing new line characters, punctuation, single character-words and performing spell checks [14]. A recent study found that a Dutch version of this model, referred to BERTje, outperformed traditional approaches (TF-IDF and doc2vec with ML), other health domain specific Dutch transformers RobBERT and MedRobERTa.n in predicting the reasons for undertaking MRI scan from three classes: (1) Diagnosis; (2) Progression; or (3) Monitoring [15]. eXplainable Artificial Intelligence (XAI) techniques such as Shapley Additive Explanation (SHAP) and Local Interpretable Model-Agnostic Explanation (LIME) were used to generate variable importance for words that result in class predictions and were ranked by the radiologist to understand the average impact of (perhaps) valid words on predictions. Another study compared French language BERT models against a Bidirectional and Auto-Regressive Transformer (BART) based model for prediction emergency department triage status (hospitalised or discharged). Interestingly, PCA dimensionality reduction of transformer embedding was applied followed by k-means clustering of different cluster sizes ranging from 2–10 and cluster membership robustness assessed through the Silhouette score and the Fowlkes–Mallows (FM) score, suggesting models with larger number of parameters having higher performance [14].

Previous research has mostly concentrated on extracting predefined outcomes from echocardiographic records. There have only been two published studies that integrated corpus-specific knowledge for complete extraction of measurement outcomes [10,11]. Few studies have applied automated conversion of units to a standardised dictionary-specified format. In addition, few studies have clinically applied the data extraction approach within the National Health Service (NHS) settings utilising efficient EHR database extraction and data loading processes for maximising the availability of structured echo report results.

## 3. Materials and Methods

The register-based cohort study using de-identified patient data is part of a research approved by the Bristol Heart Institute, and the need for patients’ consent was waived. Reporting of results follows the TRIPOD statement.

### 3.1. Dataset

The study was performed using the University Hospitals Bristol and Weston NHS Foundation Trust (UHBW) echocardiogram dataset, which comprises UHBW echocardiogram reports prospectively stored by UHBW following patient examinations. Cardiac resynchronisation optimisation echo, 3D TTE, TTE with contrast agents; Stress Echocardiogram (SE) with and without dobutamine and contrast agents; and Trans-Oesophageal Echocardiogram (TOE) cases were excluded, resulting in a total of 135,062 TTE reports for analysis. This study was performed using data from all TTE echocardiogram examinations across the UHBW from January 2009 to November 2020. A total of 200 routinely reported echocardiography outcomes measurements were extracted by the system at baseline. All names and demographics were removed from the echocardiography reports and corresponding metadata.

Although obtaining true labels for examination results through manual annotation can be a laborious process, two cardiologists undertook the task of blindly labelling of 98 reports from this TTE cohort to extract examination results for 43 of the most clinically relevant (35 continuous and 8 discrete) outcomes as the gold standard for the test dataset (Table 1). Differences in extraction were resolved by the lead cardiologists. Variables extracted by the cardiologists was on the same unit scale as that within the original echocardiography report.

The dataset was split into three cohorts: Training and Validation (n = 1075), Test Dataset (n = 98) and Application Dataset (n = 133,889). The primary evaluation criteria were discrimination, calibration, and overall accuracy of the NLP system in the extraction of TTE echocardiogram variables into the structured format.

### 3.2. Data Exploration

A list of common occurring words, but which are not specifically echocardiogram variable related, in the echocardiographic examination reports was curated (see Supplementary Materials Figure S1). This list was entered into the high dimensionality Cluto clustering toolkit as exclusion criteria algorithm, along with the training/validation dataset to identify important variables within the current context [16]. Hierarchical clustering was utilised to cluster variables that showed a high degree of similarity across documents.

The clustering was analysed using (i) automatic clustering (Figure S2), allowing the algorithm to determine the appropriate detailed level of the clustering structure; and (ii) using a clustering size of 3 to show higher level similarities across variables (Figure S3). Due to the high dimensionality of the heatmap, Ghostscript interpreter was used to visualise the heatmap. This exploratory analysis, along with echocardiography expertise was used to guide the categorisation of system extraction components including the JAPE rules (as discussed later).

An analysis of variations across echocardiogram variables was then conducted for all 200 outcome variables (Table 2). This process was supported by the use of Regular Expressions within macro based excel spreadsheets to rapidly establish presence and location of each variable. The underlined textual elements show features that are taken into account during the text annotation and extraction process.

### 3.3. Data Annotation Approach

In order to capture crucial information such as heart dimensions, ejection fraction, valve properties and other relevant measurements, we first defined specific data categories in preparation for the annotation process. These categories were designed to ensure the comprehensive extraction and standardisation of both discrete and continuous variables. This annotation process adhered to the UK echosonographers guidelines, as well as categories established in collaboration with medical experts [17].

NLP-based annotations were performed using the JAPE engine, which automatically annotated data from echocardiography reports. The annotation pattern description is situated on the left-hand side (LHS) of the JAPE, whereas the annotation manipulation statements are found on the right-hand side (RHS) [18]. The annotations identified on the LHS were then processed on the RHS using labels from the LHS to refer to matched text segments. RHS processing tasks included unit conversions, the conversion of qualitative data to binary or ordinal formats, the addition of annotations (units and measurements values (varValue)) for the matched patterns and grouping annotations to higher parent-ontological levels (e.g., Sinotubular Junction is grouped under parent level VesselsMeasurements). The annotations and corresponding measurement varValues are then parsed by the JAVA functions for conversion into structured CSV (comma-separated values) format before insertion into the designated SQL database tables, according to the parent-ontological levels.

Two cardiologists separately performed manual annotations in excel (CSV) spreadsheets to enable the validation of the correctness and reliability of the NLP generated annotations. For both NLP and clinical based CSV annotations, we collapsed the outcome columns in from wide to long formats to enable metric-based performance evaluations.

### 3.4. Model Development

**Figure 1:**
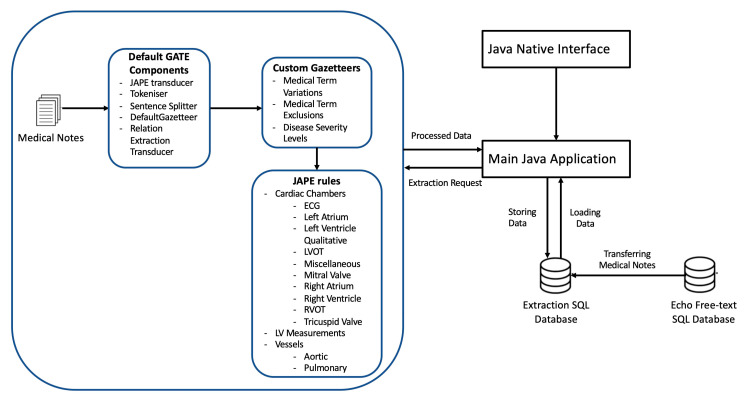
Design of the NLP Echo extraction system.

The model was developed with the Java and GATE NLP framework using the Eclipse development environment. The JAPE Transducer was included to enable mapping of the input text to output text, in order to enable other components to be integrated as part of the processing pipeline. We used the GATE tokeniser, which separates the input text into discrete tokens that represent individual meaningful language components, such as words or punctuation. The sentence splitter was then used to separate the text into individual sentences based on sentence boundaries identified by the output of the tokeniser (Figure 1).

We then used the Java Annotation Patterns Engine (JAPE, as explained further later on) to establish unique annotation rules over the text. We were able to define and match particular token patterns (combinations) using JAPE rules, applying new annotations based on the patterns discovered.

In addition, we used gazetteers [19], referred to herein as dictionaries, which are standardised groupings of terms or phrases associated with a specific topic. Gazetteers contributed to improving the tokenisation process by ensuring proper treatment of echocardiography terms, levels of severity and exclusion topics that could not have been adequately captured in the general language corpora or tokeniser. The JAPE rules and dictionaries were developed for 200 echocardiographic examination outcomes.

Furthermore, our technique is cross data repository compatible, enabling direct extraction to any specified data repository within the same distributed NHS network.

JAPE is a rule-based language that is used in GATE to extract information from text. JAPE rules provide patterns and actions for detecting linguistic patterns in the text and create or modify annotations [19]. These rules are carried out in a sequential manner, specified by their priority to resolve overlapping annotations. GATE JAPE rules were further used to extract the corresponding numerical (integer or float) or qualitative categorisation values of each variable. Some examples are provided in Table S1.

For example, the aortic regurgitation (AR) level was defined quantitatively as integers with the following criteria: 0—no regurgitation; 1—Trivial regurgitation; 2—Mild regurgitation (Or Trivial–Mild); 3—Moderate regurgitation (Or Mild–Moderate); 4—Severe regurgitation (Or Moderate–Severe), while Left Ventricular (LV) Systolic Function were defined qualitatively with the following criteria: Hyperdynamic, Normal, Borderline, Mild, Mild–Moderate, Moderate, Moderate–Severe, Severe. For continuous values that have a range scale, e.g., Ejection Fraction (EF) 45–50%, the system extracted the average of the range values (47.5%).

Apart from the ability to pattern match data variations, the JAPE rules were designed to extract numerical clinical measurement values (Table S1). During this process, our technique automatically converts any measurement values into pre-defined scales and units, standardising to a common unit scale where appropriate. For example, an outcome reported in centimetres (cm) in one examination but in meters (m) in another would have been standardised to cm by multiplication of 100 in the second report, if cm was the most commonly reported unit.

**Figure 2:**
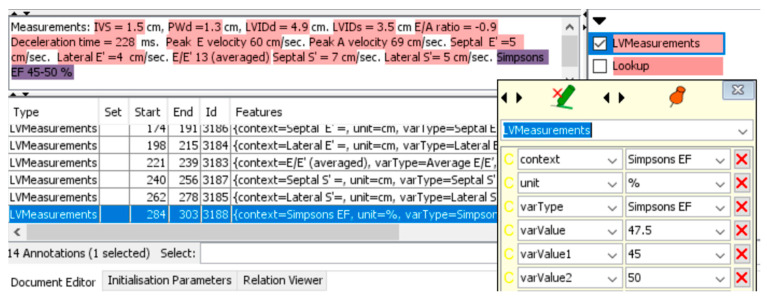
Graphical user interface of system to explainability of extraction for the continuous variable, EF. varValue1 shows the lower range value if this exists; varValue2 shows the upper range value; varValue shows the average of lower and upper range values if these exist.

A graphical user interface (GUI) option can be turned on in the system to enable real-time tracking of the extraction process and to provide explainability of the extraction process (Figure 2). Due to increased memory consumption and processing time, this function was turned on during the training and validation phase but was switched off during the test and application phases to enable annotations at high throughput. Red highlighted sections of the report show the relationship annotations generated through the JAPE rules for associating outcomes with their measurements.

The JAVA interface then enables the extraction of these annotations for matching and inserting into the corresponding table columns in the Microsoft SQL server database. The NLP model was iteratively refined using the training and validation dataset using echocardiography expert knowledge to drive human in the loop reinforcement-based improvements. Microsoft SQL server database was used for storing and loading raw and extracted reports through an interface with the main Java application.

### 3.5. Statistical Analysis and Validation

The results for the aggregated mean and standard deviation of each continuous variable for both the doctor and NLP extractions were reported in the exploratory and calibration analysis. Additionally, coefficients of determination (R2) and intraclass correlation coefficients (ICC) [13,20,21,22] with *p*-value for continuous variables were calculated. ICC is a measure of interrater reliability and was categorised as poor: <0.5; moderate: 0.5–0.75; good: 0.75–0.9 and excellent: >0.9 [23].

The magnitude calibration of the variable values in the dataset was visualised using bubble plots. The x and y positions in these plots were determined by the total (combined) magnitude of the values across all test dataset reports for each outcome, and the size of the circles indicated how frequently the variable values occurred.

For the performance evaluation of discrete variables, we adopted the confusion matrix, precision-recall and the F1 score. The confusion matrix was determined to evaluate the system’s overall accuracy, while precision and recall values were applied to evaluate how accurately the system identified positive cases (presence of measurements), while avoiding false positives (measurement extracted where not actually present) and false negatives (measurement missed in the extraction process), respectively. Rare outcomes were aggregated to prevent undefined (NaN) values that would otherwise prevent the calculation of precision and recall values. F1 score was utilised for evaluation of the overall performance of positive case extraction.

### 3.6. Practical System Application

Once development was complete, the system was installed onto the central hospital server and utilised to process the automated extraction for 133,962 separate TTE reports (Application Dataset).

## 4. Results

### 4.1. Demographics

A total 146,967 echocardiographic examinations were conducted on 78,536 patients within UHBW during the study period at baseline. Cardiac resynchronisation optimisation echo, 3D TTE, TTE with contrast agents; SE with and without dobutamine and contrast agents; and Trans-Oesophageal Echocardiogram (TOE) cases were excluded, resulting in 135,062 reports. The pre-processing of data has been described previously in the methods section. A patient flow consort diagram is shown in Supplementary Materials Figure S4. Baseline differences in echocardiogram variables between clinician and algorithm extractions are shown in Table 3.

Using coefficients of determination (R^2^) and intraclass correlation coefficients (ICC) for continuous outcomes, the effectiveness of the system for outcome measures extraction was assessed (Table 4). Variables including LVOT VTI, LVOT Vmax, AV VTI and TR Vmax showed R^2^ values of 1, demonstrating a perfect fit between the predictions of the system and the clinicians’ extractions. Of these outcomes, LVOT VTI, AV VTI and TR Vmax exhibited perfect ICC scores (1.00, *p* < 0.05) alongside high R^2^ values, demonstrating ideal agreement between the system and clinicians.

The ICC results demonstrated a good level of inter-rater reliability for numerous outcomes. ICC values of 0.75–0.9 were observed for variables such as LVOT Diam, Lateral MAPSE, Peak E Velocity, Lateral E’ Velocity, PV Vmax, Sinuses of Valsalva and Ascending Aorta diameters. Furthermore, with excellent ICC scores (0.97 ^b^, 0.99 ^b^, 0.92 ^b^ and 0.90 ^b^, respectively) and high R^2^ values, the outcomes LA Area, LA Volume, RA Area and Peak A Velocity demonstrated excellent inter-rater reliability across the system and clinicians’ extractions.

**Figure 3:**
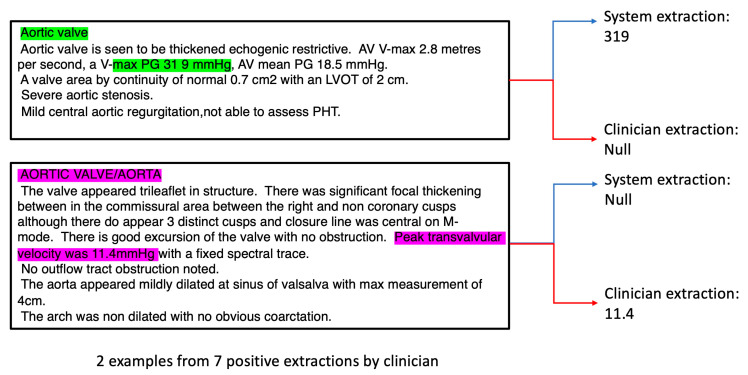
In depth analysis of clinician and system extractions for AV max PG; green and pink region indicates region of features involved in the annotation process.

For outcomes with very low R and ICC values e.g., Aortic Valve max Pressure Gradient (AV max PG), we analysed the reports in detail and discovered that the reason for low observed performance was due to the following factors: (i) the system extracted the correct values, but the clinicians missed such values (Figure 3, green annotations), possibly due to incorrect recording by the echocardiographer. In this case, the “38 9” should likely be recorded as “38.9”, instead when considering the correct scale required; (ii) the echocardiographer used the wrong terminology (e.g., incorrectly using peak transvalvular velocity to describe AV max PG, Figure 3, purple annotations);

(iii) due to low number of cases, there were only 7 AV max PG positive cases recorded by the clinician out of 98 documents in the test dataset. Hence, non-system induced errors (e.g., from (i) and (ii)) can inflate the perceived underperformance of the system.

**Figure 4:**
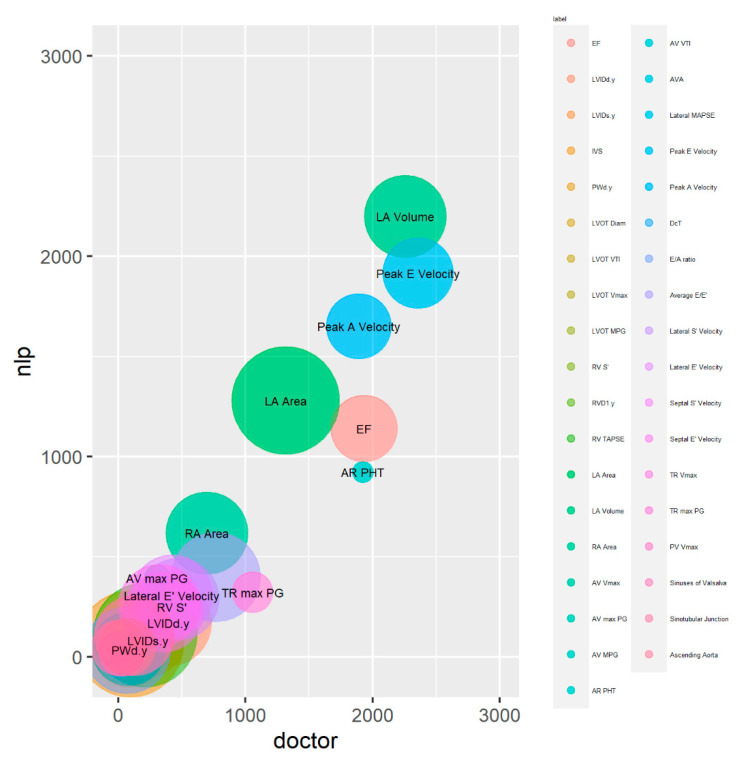
Bubble plot analysis of magnitude and calibration for continuous variable sets. *X*-axis and *y*-axis show the total magnitude of extracted outcome measures for clinician and NLP, respectively. The size of circles represents the frequency of each variable extracted by the NLP system.

In the bubble plot, larger bubbles represent frequently occurring outcomes across all reports in the test dataset, while smaller bubbles represent less frequent outcomes (Figure 4). The bubble plot demonstrates that the LA area, located close to the plot’s centre, is represented by the largest circle. This shows that the LA area is the variable that is most frequently extracted. Other variables with substantial circle sizes include LA volume, Lateral E’ velocity and RA area, indicating that they are also frequently extracted by the NLP system.

The centres of circles of the bubbles in the bubble plot are closely aligned to the diagonal line from the origin, indicating that the system and clinician extractions are well calibrated overall. However, certain variables, including EF, AR PHT and TR max PG, were below the diagonal line, suggesting under-extraction by the system compared to the clinician.

Using precision, recall and F1 score metrics, we assessed the effectiveness of the system for extracting discrete outcome measures (Table 5).

### 4.2. Aortic Regurgitation (AR) Level

The system showed a precision of 0.86 and a recall of 0.89 for the identification of AR levels. Only three occurrences (false negatives) were missed while accurately identifying 24 true positive situations. The system generated four instances of false positives. The final F1 score was 0.87, demonstrating a performance that was balanced across recall and precision.

### 4.3. LV Systolic Function

The NLP algorithm achieved a precision of 0.80 and a recall of 0.41 for the classification of Left Ventricular (LV) Systolic Function. While missing 17 occurrences (false negatives), it accurately recognised 12 true positive cases. Three false positive cases were produced by the algorithm. In this situation, there was a trade-off between precision and recall, as indicated by the F1 score of 0.55.

### 4.4. MV Regurgitation Level

The NLP algorithm performed exceptionally in the detection of mitral valve (MV) regurgitation levels. With a precision and recall of 0.98 and 0.97, respectively, it was able to correctly identify 59 true positive cases while producing only two false negatives. One false positive case was obtained by the algorithm. The final F1 score was 0.98, which represents a strong overall performance.

### 4.5. AV+MV+PV+TV Stenosis

The aortic valve (AV), mitral valve (MV), pulmonic valve (PV) and tricuspid valve (TV) stenotic levels that were extracted were aggregated, since occurrences were rare and were classified with moderate precision and recall by the system. The precision and recall were 0.38 and 0.56, respectively. Five true positive cases were accurately recognised by the algorithm, whereas four false negative cases were missed. It produced eight instances of false positives. The F1 score for this result was 0.45, indicating a performance that was reasonably balanced between recall and precision.

### 4.6. TR Level

The system displayed high precision and moderate recall for classifying extractions of tricuspid regurgitation (TR) levels. It achieved a precision of 0.97 and a recall of 0.64. A total of 32 false negatives were missed by the system, while 57 true positive cases were successfully identified. It obtained two false positive cases. The F1 score for this result was 0.77, indicating a favourable balance of precision overall.

**Figure 5:**
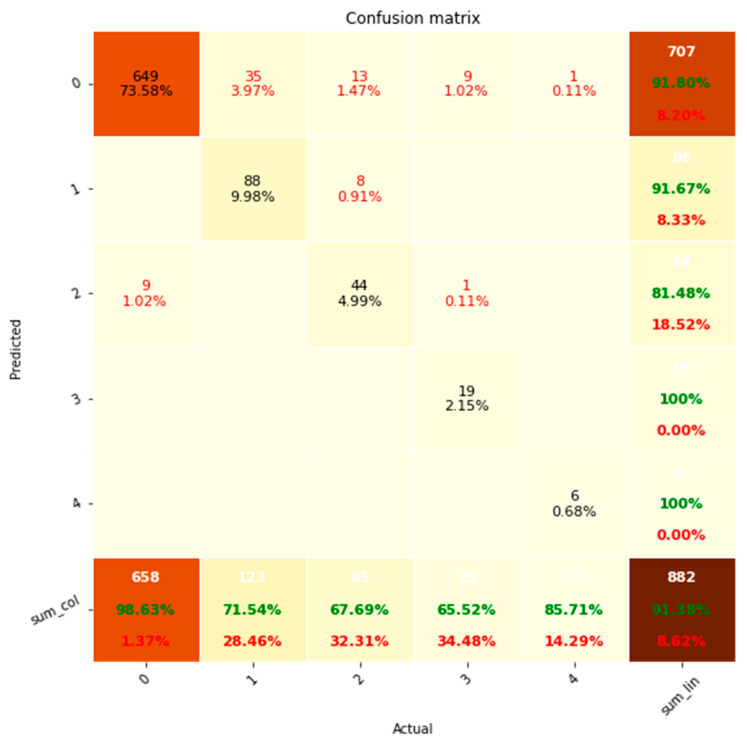
Confusion matrix analysis of classification performance for discrete variable sets. system (Predicted) is shown on the *y*-axis while the clinicians (Actual) is shown on the *x*-axis. Accuracies are shown in green and errors in red. % represents percentage of total.

The confusion matrix exhibits a predominant pattern where the extractions align diagonally from the top left to the bottom right (Figure 5), indicating a high degree of correct classification. Out of the 882 instances evaluated, the NLP system achieved an accuracy rate of 91.38%. The remaining 8.62% of errors primarily consisted of false negatives, accounting for 7.6% of the total. Conversely, false positives constituted only 1.02% of the errors.

## 5. Discussion

This study presents the use of a GATE-based system for extracting outcomes from TTE reports, with a special emphasis on capturing both discrete and continuous variables. Most of the rule-based research in the field of NLP in healthcare have focused on general clinical text, methodology or specific medical domains, with limited exploration in the context of echocardiography reports or the use of GUI based interface to interpret extraction [18,20,21,22,24,25,26,27,28]. To the best of our knowledge, this is one of the first studies to use a GATE-based NLP system for TTE extraction, adding to the understanding of natural language processing (NLP) in this area.

Our study set out to achieve two separate aims that aligned to increase the management and utility of echocardiographic data. First, we attempted to create a moderate-fidelity echocardiography report database by utilising automated data extraction and curation. The intention to integrate this with high-fidelity datasets, exemplified in [6], highlights the potential of multimodality learning strategies [7].

Our analysis also included identifying outcomes with consistently high extraction performance. The selection of robust variables is paramount since it provides the foundation for potential clinical applications and the development of predicted risk scores. These two initiatives are aligned, aiming to improve data utilisation, ultimately enabling more efficient use of echocardiographic data in both research and clinical contexts. 

### 5.1. Technical Perspective

While studies have generally evaluated outcomes irrespective of the data type, this study presents an evaluation pipeline for considering discrete and continuous outcomes using their own respective sets of evaluation metrics. A recent study demonstrates that, for both discrete and sequential datasets, discrete-feature approaches outperformed sequential time-series (continuous variable) methods [29]. This finding contrasted with conventional assumptions and emphasised the importance of integrating discrete data, which is consistent with our exploration of diverse variable types in echocardiography reports. Another study highlighted the advantages of translating datasets into appropriate formats, by comparing continuous (visual analogue scales) against discrete (verbal descriptor scales) rating scales [30]. It was found that both continuous and discrete rating scales gave similar performances in terms of inter-rater reliability. However, raters were found to prefer the continuous scale. This is consistent with our work to efficiently classify and handle data from echocardiogram reports using either continuous or qualitative rating scales, when and where appropriate.

In relation to our NLP-system study, applying insights from the broader deep learning domain’s debate on merging discrete and continuous processing has important implications, as indicated in [31]. The inherent role played by discrete symbols in human communication coincides nicely with the language of medical reports and allows for nuanced comprehension of discrete outcome rating scales. The idea that discrete symbols need context-expansion by incorporating continuous scale outcomes resonates with the explanative nature of the latter type of data. This data framework of integrating discrete and continuous data parallels the structured data generated from the NLP system in this study.

There are numerous methods to mix continuous and discrete processing in ways that support gradient-based learning in addition to attention mechanisms. It is common practise to use policy gradients [32], a technique for backpropagation through discrete decisions [33]. A differentiable method for choosing distinct categories in a sampling setting is provided by reparameterisation techniques such as the Gumbel–Softmax distribution [34]. Modelling a categorical probability distribution over the discrete elements, determined by the continuous input, is also a popular technique for converting continuous representations into discrete outputs (Bengio et al., 2003) [29,35]. Discrete elements (such as word tokens or actions) can be transformed into continuous representations by locating the appropriate token (feature) vector from its matrix embeddings using the corresponding token index.

It has also been suggested that composable neural module networks (NMN) might be advantageous for combining continuous and discrete information [36,37]. These modules can be combined into intricate networks according to the computations required to respond to natural language queries. They are specialised for specific subtasks. By concentrating on training component modules and learning how to combine them, this method minimises the need to relearn for every type of issue. The JAPE rules as part of the JAVA interfacing NLP system in this study have similarities in principle to the methodology for composing NMNs modules [38], and may provide the basis for integrating neural based approaches to TTE extraction systems in an interpretable manner. For studies have generally focusing on image based problems, the attention module generally prefers the use of attention extracted from Convolution Neural Networks [39,40]. Recurrent and Transformer Neural Networks may be more suitable to model text and non-text based sequential interactions [41], with the latter able to excel in modelling long term dependencies, through parallel processing and self-attention to capture relationship across input all input features simultaneously.

### 5.2. Relevance to Clinical Practice

#### 5.2.1. Cardiac Surgery Perspective

In addition to its applications within the context of echocardiography reports, the NLP system may have potential for broader clinical implications and research opportunities. Preoperatively, the algorithm offers the possibility of identifying critical parameters from echo data that could act as “red flags”, prompting surgeons to prioritise surgery for patients with specific aortic diameters or valve haemodynamics. This functionality may assist surgeons in managing surgery waiting lists more efficiently.

Following surgical interventions, the system’s functionalities may extend to the post-operative phase, assisting clinicians to promptly identify issues related to structural valve degeneration and adjust the frequency of monitoring accordingly. Furthermore, the algorithm may play a potential role in addressing challenges like patient-prosthesis mismatch, enabling clinicians to make informed decisions for clinical interventions and facilitating audits for quality control purposes.

Increasingly, congenital heart disease (CHD) is diagnosed in the antenatal setting, with foetal echocardiography commonplace. Extension of the system’s functionalities to antenatal ultrasound imaging modalities could aid antenatal risk modelling and prognostication, facilitating improved surgical planning, perinatal and neonatal care.

The system’s applicability includes not only echocardiography report analysis but also other types of investigative modalities. For instance, the system’s capabilities might be increased to examine computer tomography (CT) results and aid in aortic surveillance initiatives. Additionally, the system holds potential to integrate risk modelling approaches with automated text extraction, while identifying risks that have the potential for advancing research projects in cardiac care.

#### 5.2.2. Cardiologist Perspective

The ability to extract echo data for reports using NLP would have significant benefits to managing a range of patients, including those with valvular heart disease, heart failure and ischaemic heart disease. As workflows in clinical practice become more automated, it is crucial to be able to prioritise and manage referrals and outpatient waiting lists. Being able to identify patients who have had a significant worsening of their valvular heart disease or cardiac function in a way that does not increase the administrative burden of reviewing every echo report would be extremely appealing to cardiologists. This tool also has great potential for research and auditing. For instance, with the ability to track the rates of change of aortic stenosis over time or monitoring aortic root dimensions. In addition, the combination of this database with other biochemistry, chest imaging and electrocardiogram datasets holds potential for the improved diagnosis of heart failure in patients.

This tool can integrate both the quantitative and qualitative data from an echo report, which is crucial for the optimal management of these patients.

### 5.3. Limitations and Future Work

Future work shall investigate the comparison of ICD-10 diagnostic codes against NLP for detecting cardiac diseases in the evaluation of this patient cohort [9]. Due to the cost in terms of time and effort required for clinicians to manually annotate and extract the validation labels, a limited number of evaluation samples were available. Thus, future work should seek to obtain a larger sample of labelled validation samples from the entire hospital extracted set. It would also be interesting to refine the categorisation process to capture more nuanced information from echocardiography reports. This could involve identifying and extracting specific details related to different cardiac conditions e.g., heart failure. Future work should also further improve the NLP system compatibility with existing electronic health record systems to seamlessly integrate with healthcare IT infrastructure for wider adoption, possibly with a web-based view of the extracted data to enable echocardiographers or clinicians to perform quality inspections and manually modify the extracted data. Since manual annotations by clinicians using Excel spreadsheets were laborious, future work may consider the possibility of rapid annotation approaches whereby the NLP pre-annotates the reports in the GUI environment to allow the clinicians to only make minor corrections before curating a high fidelity dataset. This approach may also help to rapidly generate large amounts of gold standard annotations for the evaluation of newly developed transformer-based tools, or for the purpose of mechanistic clinical studies on the high fidelity corrected dataset. The application of Cluto-based clustering using graph representations is possible but was not investigated. This, and similar approaches, would be interesting to analyse in future studies [16,42]. While the approach has also been developed for extracting Magnetic Resonance Imaging (MRI) unstructured data, future work should identify clinicians with expertise for curating the validation labels in this type of device. Certain variables, such as LVOT Vmax, showed high R^2^ correlation but low ICC scores, suggesting that the scalar value of the NLP system extraction is correct but potentially on a different unit scale to that extracted by the clinician. Future work should aim to develop a plugin to the existing system to convert the clinician extraction to the NLP system unit scale. The mean values for some continuous outcomes extracted by the system were lower than those by the cardiologists, suggesting that any differences are more likely due to false negative than false positive findings. Although any improvements in extraction process would still be limited by the accuracy of the initial report, standardising the reporting of echocardiography across different vendors, national and international societies would be beneficial. Future work should also aim to further develop the approach using hybrid (machine learning plus rule-based) approaches to enhance existing variables with relatively lower extraction performance. For example, the combination with NMN-based approaches through reinforcement learning to select modules may encapsulate greater variations in outcome terminologies across different hospital locations. As another possible direction for future work, more advanced NLP models (e.g., BERT) could be utilised for the text analysis task. BERT models offer the advantage of contextual understanding through embedding representations, allowing them to capture intricate relationships within text and to enhance the accuracy and depth of the analysis. It would also be interesting to incorporate fuzzy [10], foundation learning and reinforcement based approaches on a larger local validation cohort to further improve the modelling performance. Future work should also consider analysing data extracted from echocardiography reports over time to track disease progression and treatment outcomes. One should also aim to validate the NLP system’s performance in diverse patient populations, to ensure that it can effectively handle variations in language and medical terminology.

## 6. Conclusions

In summary, this study offers a novel application of the GATE-based system in the extraction of echocardiogram report data to curate a moderate fidelity TTE database that could be used for multi-fidelity data fusion. The study contributes to the understanding of NLP in the context of echocardiography by addressing both approaches for discrete and continuous outcomes. These findings emphasise the valuable contribution of NLP in automating data extraction and improving clinical decision-making processes. By bridging the gap between structured and semi-structured healthcare data, our approach holds promise for advancing research, risk prediction and patient care in the realm of echocardiography and beyond.

**Guarantor TD.**

**Code Availability:** Code for the system is available on GitHub: https://github.com/s0810110/neoImage/tree/neoImage_v-1.1 (accessed on 2 August 2023) and validation: https://github.com/s0810110/neoImageClinicalValidation (accessed on 1 November 2023); access can be provided upon reasonable request with the main author. Analyses were performed using JAVA.

## Figures and Tables

**Table 1:** Abbreviations and definitions for 43 of the most clinically relevant outcomes labelled by the two clinicians. Heart category and data type are also shown.

Abbreviation Clinician	Abbreviation NLP	Definition	Category	Data Type
LVEF	EF	Left Ventricular Ejection Fraction	**Left ventricle**	Continuous
LVIDd	LVIDd	Left Ventricular Internal Diameter in Diastole		Continuous
LVIDs	LVIDs	Left Ventricular Internal Diameter in Systole		Continuous
LVSF	LV Systolic Function	Left Ventricular Shortening Fraction		Discrete
IVSd	IVS	Interventricular Septum Thickness in Diastole		Continuous
PWd	PWd	Posterior Wall Thickness in Diastole		Continuous
LVOTd	LVOT Diam	Left Ventricular Outflow Tract Diameter		Continuous
LVOTvti	LVOT VTI	Left Ventricular Outflow Tract Velocity-Time Integral		Continuous
LVOTpv	LVOT Vmax	Left Ventricular Outflow Tract Peak Velocity		Continuous
RV_TDI_S	RV S’	Right Ventricle Tissue Doppler Imaging S Wave	**Right ventricle**	Continuous
RVD1	RVD1	Right Ventricle Diameter at Basal Level		Continuous
TAPSE	RV TAPSE	Tricuspid Annular Plane Systolic Excursion		Continuous
LA_area	LA Area	Left Atrial Area	**Left atrium**	Continuous
LA_vol	LA Volume	Left Atrial Volume		Continuous
RA_area	RA Area	Right Atrial Area	**Right atrium**	Continuous
AS_sev	AV Stenosis	Aortic Stenosis Severity	**Aortic valve**	Discrete
AR_sev	AR level	Aortic Regurgitation Severity		Discrete
AVpv	AV Vmax	Aortic Valve Peak Velocity		Continuous
AVpg	AV max PG	Aortic Valve Peak Gradient		Continuous
AVmg	AV MPG	Aortic Valve Mean Gradient		Continuous
AVpht	AR PHT	Aortic Valve Pressure Half-Time		Continuous
AVvti	AV VTI	Aortic Valve Velocity-Time Integral		Continuous
MS_sev	MV Stenosis	Mitral Stenosis Severity	**Mitral valve**	Discrete
MR_sev	MV Regurgitation Level	Mitral Regurgitation Severity		Discrete
MAPSE	Lateral MAPSE	Lateral Mitral Annular Plane Systolic Excursion		Continuous
MV_E_vel	Peak E Velocity	Mitral Valve E Wave Velocity		Continuous
MV_A_vel	Peak A Velocity	Mitral Valve A Wave Velocity		Continuous
MV_decT	DcT	Mitral Valve Deceleration Time		Continuous
MV_Earatio	E/A ratio	Mitral Valve E/A Ratio		Continuous
MV_EE_avg	Average E/E’	Mitral Valve E/E’ Average		Continuous
TDI_lat_S	Lateral S’ Velocity	Tissue Doppler Imaging Lateral S Wave		Continuous
TDI_lat_E	Lateral E’ Velocity	Tissue Doppler Imaging Lateral E Wave		Continuous
TDI_sep_S	Septal S’ Velocity	Tissue Doppler Imaging Septal S Wave		Continuous
TDI_sep_E	Septal E’ Velocity	Tissue Doppler Imaging Septal E Wave		Continuous
TS_sev	TV Stenosis	Tricuspid Stenosis Severity	**Tricuspid valve**	Discrete
TR_sev	TR level	Tricuspid Regurgitation Severity		Discrete
TR_pv	TR Vmax	Tricuspid Regurgitation Peak Velocity		Continuous
TR_pg	TR max PG	Tricuspid Regurgitation Peak Gradient		Continuous
PS_sev	PV Stenosis	Pulmonary Stenosis Severity	**Pulmonary valve**	Discrete
PV_Vmax	PV Vmax	Pulmonary Valve Maximum Velocity		Continuous
AO_SOV	Sinuses of Valsalva	Sinus of Valsalva	**Aorta**	Continuous
AO_STJ	Sinotubular Junction	Aortic Outflow Sinotubular Junction		Continuous
AO_ASC	Ascending Aorta	Aortic Outflow Ascending Aorta		Continuous

**Table 2:** Example of outcome measure variations for continuous and qualitative (discrete) measurements.

Aortic Valve (AV) Velocity Time Integral (VTI)	AV Regurgitation Level
AV Vmax 4.2 m/s MPD 46 mmHg VTI 103 cm	Aortic Valve (biological AVR): AVR in situ, well seated. … No significant regurgitation. … Aorta:
AV Vmax 4 m/s MPD 42 mmHg VTI 87.9 cm	Aortic Valve (unclear imaging of AVR): … AVR seen in situ with a mild paraprosthetic regurgitation. … Aorta:
Ao VTI 36 cm; AVA (VTI) 1.8 cm^2^	Aortic Valve: … with valve type/size in situ No aortic regurgitation Right Ventricle:
Ao VTI 36 cm;	Aortic Valve: … No aortic stenosis. Trivial aortic regurgitation. AV Vmax: 1.6 m/s. Aorta:
Aortic Valve: Appears Trileaflet. Thickening of LCC/NCC with reduced mobility of these cusps. V max 2.8 m/s, PPD: 32 mmHg, MPD: 19 mmHg, VTI: 62.3 cm.	Aortic Valve (TAVI): … No aortic stenosis/obstruction indicated. Trivial–mild paravalvular aortic regurgitation. … Aorta:
AV Vmax 4 m/s MPD 42 mmHg VTI 87.9 cm LVOT 2.7 cm Peak V = 0.7 m/s, MPD 1.2 mmHg, VTI 16.2 cm	Aortic Valve: AVR in situ. …No significant obstruction/stenosis indicated. No obvious aortic regurgitation. … Aorta:
AV Vmax: 4.8 m/s, PPD: 91 mmHg, MPD: 57 mmHg, VTI: 107 cm	Aortic Valve: … Mild eccentric paravalvular aortic regurgitation seen. Aorta:
AV mean PG: 54 mmHg. AV VTI: 78 cm. AVA VTI: 0.88 cm^2^. AVAi VTI: 0.45 cm/m^2^	Aortic Valve: …. Mild transvalvular aortic regurgitation. …
AV VTI 61.3 cm	Aorta: Aortic Valve: … Aortic regurgitation present …Overall assessment is of mild aortic regurgitation. AV Vmax: 1.5 m/s. Aorta:
Ao VTI 106 cm;	Aortic Valve: … ? BAV. Moderate AS. Trivial AR …. Aorta:
AV VTI: 78 cm.	Aortic Valve: …. Overall assessment is of severe aortic regurgitation. … Aorta:

**Table 3:** Baseline patient demographics.

	Baseline Patient Total = 78,536
Age (years), mean (SD)	59.4 (33.0)
Female gender, n (%)	36,959 (47.1%)
Mortalities, n (%)	25,048 (31.9%)
Number of repeat echocardiograms	
1	53,508 (68.1%)
2	13,031 (16.6%)
3	5064 (6.4%)
4	2675 (3.4%)
5	1632 (2.1%)
6	933 (1.2%)
7	531 (0.7%)
8	356 (0.5%)
9	233 (0.3%)
>10	573 (0.7%)

**Table 4:** Coefficients of determination (R2) and intraclass correlation coefficients (ICC) with *p*-value for continuous variables; **^a^** represents good interrater reliability 0.75–0.9; **^b^** indicates excellent interrater reliability 0.9–1.0 [23].

Variable Name	R Squared	ICC	ICC *p*-Value
EF	0.47	0.64	0.00
LVIDd	0.10	0.18	0.07
LVIDs	0.14	0.25	0.04
IVS	0.31	0.47	0.00
PWd	0.39	0.43	0.04
LVOT Diam	0.70	0.83 ^a^	0.00
LVOT VTI	1.00	1.00 ^b^	0.00
LVOT Vmax	1.00	0.02	0.42
RV S’	0.10	0.29	0.00
RVD1	0.07	0.16	0.05
RV TAPSE	0.11	0.16	0.05
LA Area	0.95	0.97 ^b^	0.00
LA Volume	0.98	0.99 ^b^	0.00
RA Area	0.86	0.92 ^b^	0.00
AV Vmax	0.13	0.29	0.00
AV max PG	0.00	0.03	0.39
AV MPG	0.20	0.35	0.00
AR PHT	0.46	0.63	0.00
AV VTI	1.00	1.00 ^b^	0.00
Lateral MAPSE	0.60	0.76 ^a^	0.00
Peak E Velocity	0.59	0.76 ^a^	0.00
Peak A Velocity	0.81	0.90 ^b^	0.00
DcT	0.05	0.15	0.06
E/A ratio	0.18	0.36	0.00
Average E/E’	0.03	0.13	0.08
Lateral S’ Velocity	0.49	0.65	0.00
Lateral E’ Velocity	0.62	0.77 ^a^	0.00
Septal S’ Velocity	0.45	0.62	0.00
Septal E’ Velocity	0.49	0.69	0.00
TR Vmax	1.00	1.00 ^b^	0.00
TR max PG	0.30	0.45	0.00
PV Vmax	0.62	0.77 ^a^	0.00
Sinuses of Valsalva	0.73	0.85 ^a^	0.00
Sinotubular Junction	0.82	0.17	0.03
Ascending Aorta	0.64	0.78 ^a^	0.00

**Table 5:** Precision recall results for discrete variables sets in the test dataset.

Outcome	TP	FN	FP	TN	Precision	Recall	F1 Score
AR level	24	3	4	67	0.86	0.89	0.87
LV Systolic Function	12	17	3	66	0.80	0.41	0.55
MV Regurgitation Level	59	2	1	36	0.98	0.97	0.98
AV + MV + PV + TV Stenosis	5	4	8	375	0.38	0.56	0.45
TR Level	57	32	2	105	0.97	0.64	0.77

## Data Availability

All data used in this study are from the UHBW and can be provided upon on reasonable request subject to regulatory approval.

## Supplementary Materials

The following supporting information can be downloaded at: https://www.mdpi.com/article/10.3390/bioengineering10111307/s1, Figure S1: Exclusion words used in Clustering visualisation; Figure S2: Automatic clustering visualisation; Figure S3: Clustering visualisation using cluster size of 3; Figure S4: Consort diagram showing flow of Echocardiogram reports through the study; Table S1: Examples of JAPE rules used for matching.

## References

[1] Thompson J., Hu J., Mudaranthakam D.P., Streeter D., Neums L., Park M., Koestler D.C., Gajewski B., Jensen R., Mayo M.S. (2019). Relevant Word Order Vectorization for Improved Natural Language Processing in Electronic Health Records. Sci. Rep..

[2] Zhang Y., Liu M., Hu S., Shen Y., Lan J., Jiang B., de Bock G.H., Vliegenthart R., Chen X., Xie X. (2021). Development and multicenter validation of chest X-ray radiography interpretations based on natural language processing. Commun. Med..

[3] Kim Y., Lee J.H., Choi S., Kim J.-H., Seok J., Joo H.J. (2020). Validation of deep learning natural language processing algorithm for keyword extraction from pathology reports in electronic health records. Sci. Rep..

[4] Morgan S.E., Diederen K., Vértes P.E., Ip S.H.Y., Wang B., Thompson B., Demjaha A., De Micheli A., Oliver D., Liakata M. (2021). Natural Language Processing markers in first episode psychosis and people at clinical high-risk. Transl. Psychiatry.

[5] Dickerson L.K., Rouhizadeh M., Korotkaya Y., Bowring M.G., Massie A.B., McAdams-Demarco M.A., Segev D.L., Cannon A., Guerrerio A.L., Chen P.-H. (2019). Language impairment in adults with end-stage liver disease: Application of natural language processing towards patient-generated health records. NPJ Digit. Med..

[6] Liu L., Zhang C., Tao D. (2023). GAN-MDF: A Method for Multi-fidelity Data Fusion in Digital Twins. arXiv.

[7] Liu Z., Yin H., Chai Y., Yang S.X. (2014). A novel approach for multimodal medical image fusion. Expert Syst. Appl..

[8] Gotz D., Borland D. (2016). Data-Driven Healthcare: Challenges and Opportunities for Interactive Visualization. IEEE Comput. Graph. Appl..

[9] Large-Scale Identification of Aortic Stenosis and Its Severity Using Natural Language Processing on Electronic Health Records—ScienceDirect. https://www.sciencedirect.com/science/article/pii/S2666693621000256.

[10] Szekér S., Fogarassy G., Vathy-Fogarassy Á. (2023). A general text mining method to extract echocardiography measurement results from echocardiography documents. Artif. Intell. Med..

[11] Nath C., Albaghdadi M.S., Jonnalagadda S.R. (2016). A Natural Language Processing Tool for Large-Scale Data Extraction from Echocardiography Reports. PLoS ONE.

[12] Kim Y., Garvin J.H., Goldstein M.K., Hwang T.S., Redd A., Bolton D., Heidenreich P.A., Meystre S.M. (2017). Extraction of left ventricular ejection fraction information from various types of clinical reports. J. Biomed. Inform..

[13] Zheng C., Sun B.C., Wu Y.-L., Ferencik M., Lee M.-S., Redberg R.F., A Kawatkar A., Musigdilok V.V., Sharp A.L. (2022). Automated interpretation of stress echocardiography reports using natural language processing. Eur. Heart J. Digit. Health.

[14] Arnaud E., Elbattah M., Gignon M., Dequen G. Learning Embeddings from Free-text Triage Notes using Pretrained Transformer Models. Proceedings of the 15th International Joint Conference on Biomedical Engineering Systems and Technologies (BIOSTEC 2022), Workshop on Scaling-Up Health-IT.

[15] Rietberg M.T., Nguyen V.B., Geerdink J., Vijlbrief O., Seifert C. (2023). Accurate and Reliable Classification of Unstructured Reports on Their Diagnostic Goal Using BERT Models. Diagnostics.

[16] Kim Y., Riloff E., Meystre S.M. (2018). Exploiting Unlabeled Texts with Clustering-based Instance Selection for Medical Relation Classification. AMIA Annu. Symp. Proc..

[17] Robinson S., Rana B., Oxborough D., Steeds R., Monaghan M., Stout M., Pearce K., Harkness A., Ring L., Paton M. (2020). A practical guideline for performing a comprehensive transthoracic echocardiogram in adults: The British Society of Echocardiography minimum dataset. Echo Res. Pract..

[18] Andrade J.C.B., Jaramillo C.M.Z. (2021). Gate-Based Rules for Extracting Attribute Values. Comput. Y Sist..

[19] Cunningham H., Tablan V., Roberts A., Bontcheva K. (2013). Getting More Out of Biomedical Documents with GATE’s Full Lifecycle Open Source Text Analytics. PLoS Comput. Biol..

[20] Yeung A., Iaboni A., Rochon E., Lavoie M., Santiago C., Yancheva M., Novikova J., Xu M., Robin J., Kaufman L.D. (2021). Correlating natural language processing and automated speech analysis with clinician assessment to quantify speech-language changes in mild cognitive impairment and Alzheimer’s dementia. Alzheimer’s Res. Ther..

[21] Rahman M., Nowakowski S., Agrawal R., Naik A., Sharafkhaneh A., Razjouyan J. (2022). Validation of a Natural Language Processing Algorithm for the Extraction of the Sleep Parameters from the Polysomnography Reports. Healthcare.

[22] Cohen A.S., Rodriguez Z., Warren K.K., Cowan T., Masucci M.D., Granrud O.E., Holmlund T.B., Chandler C., Foltz P.W., Strauss G.P. (2022). Natural Language Processing and Psychosis: On the Need for Comprehensive Psychometric Evaluation. Schizophr. Bull..

[23] Koo T.K., Li M.Y. (2016). A Guideline of Selecting and Reporting Intraclass Correlation Coefficients for Reliability Research. J. Chiropr. Med..

[24] Khalifa A., Meystre S. (2015). Adapting existing natural language processing resources for cardiovascular risk factors identification in clinical notes. J. Biomed. Inform..

[25] Yang H., Spasic I., Keane J.A., Nenadic G. (2009). A Text Mining Approach to the Prediction of Disease Status from Clinical Discharge Summaries. J. Am. Med. Inform. Assoc..

[26] Cunliffe D., Vlachidis A., Williams D., Tudhope D. (2021). Natural language processing for under-resourced languages: Developing a Welsh natural language toolkit. Comput. Speech Lang..

[27] Digan W., Névéol A., Neuraz A., Wack M., Baudoin D., Burgun A., Rance B. (2020). Can reproducibility be improved in clinical natural language processing? A study of 7 clinical NLP suites. J. Am. Med. Inform. Assoc..

[28] Amato F., Cozzolino G., Moscato V. (2019). Analyse digital forensic evidences through a semantic-based methodology and NLP techniques. Futur. Gener. Comput. Syst..

[29] Drousiotis E., Pentaliotis P., Shi L., Cristea A.I. Balancing Fined-Tuned Machine Learning Models Between Continuous and Discrete Variables—A Comprehensive Analysis Using Educational Data. Proceedings of the International Conference on Artificial Intelligence in Education.

[30] Belz A., Kow E. (2011). Discrete vs. continuous rating scales for language evaluation in NLP. Proceedings of the 49th Annual Meeting of the Association for Computational Linguistics: Human Language Technologies: Short Papers—Volume 2.

[31] Cartuyvels R., Spinks G., Moens M.-F. (2021). Discrete and continuous representations and processing in deep learning: Looking forward. AI Open.

[32] Sutton R.S., McAllester D., Singh S., Mansour Y. (2023). Policy Gradient Methods for Reinforcement Learning with Function Approximation. Advances in Neural Information Processing Systems.

[33] Hu R., Andreas J., Rohrbach M., Darrell T., Saenko K. (2017). Learning to Reason: End-to-End Module Networks for Visual Question Answering. arXiv.

[34] Maddison C.J., Mnih A., Teh Y.W. The Concrete Distribution: A Continuous Relaxation of Discrete Random Variables. Proceedings of the 5th International Conference on Learning Representations, ICLR 2017.

[35] Bengio Y., Ducharme R., Vincent P., Jauvin C. (2001). A Neural Probabilistic Language Model. Advances in Neural Information Processing Systems 13 (NIPS 2000).

[36] Johnson J., Hariharan B., Van Der Maaten L., Hoffman J., Fei-Fei L., Zitnick C.L., Girshick R. Inferring and Executing Programs for Visual Reasoning. Proceedings of the 2017 IEEE International Conference on Computer Vision (ICCV).

[37] Andreas J., Rohrbach M., Darrell T., Klein D. Learning to Compose Neural Networks for Question Answering. Proceedings of the 2016 Conference of the North American Chapter of the Association for Computational Linguistics: Human Language Technologies.

[38] Hu R., Andreas J., Darrell T., Saenko K. (2021). Explainable neural computation via stack neural module networks. Appl. AI Lett..

[39] Mascharka D., Tran P., Soklaski R., Majumdar A. Transparency by Design: Closing the Gap Between Performance and Interpretability in Visual Reasoning. Proceedings of the 2018 IEEE/CVF Conference on Computer Vision and Pattern Recognition (CVPR).

[40] Yi K., Wu J., Gan C., Torralba A., Kohli P., Tenenbaum J. (2023). Neural-Symbolic VQA: Disentangling Reasoning from Vision and Language Understanding. Advances in Neural Information Processing Systems.

[41] Peng B., Alcaide E., Anthony Q., Albalak A., Arcadinho S., Cao H., Cheng X., Chung M., Grella M., Kiran K. (2023). RWKV: Reinventing RNNs for the Transformer Era. arXiv.

[42] Karypis G. CLUTO—A Clustering Toolkit. Report, Apr. http://conservancy.umn.edu/handle/11299/215521.

