# Supplementary material for: Development and Evaluation of a Natural Language Processing System for Curating a Trans-Thoracic Echocardiogram (TTE) Database
Source: Bioengineering (Basel). 2023 Nov 10;10(11):1307. doi: 10.3390/bioengineering10111307 (PMC10669818; doi:10.3390/bioengineering10111307)
Supplement: Supplementary file 1 [file bioengineering-10-01307-s001.zip › bioengineering-2659195-supplementary.pdf]

Supplementary Materials: Development and Evaluation of a Natural Language Processing System for curating a Trans-Thoracic Echocardiogram (TTE) database

Tim Dong, Nicolas Sunderland, Angus Nightingale, Daniel P Fudulu, Jeremy Chan, Ben Zhai, Alberto Freitas, Massimo Caputo, Arnaldo Dimagli, Stuart Mires, Mike Wyatt, Umberto Benedetto and Gianni D. Angelini.

obvious echocardiography rest rate tier sec cm2 mmhg cm2 review hears note noted notes visualise find stress Advanced clinical specialist echo Technical Quality Fair ECG GE Vivid E95 Operator study focus exercise Find research Hears Note Notes consist studies standard demonstrated demonstrate demonstrates Focused Focus heart exercises exercised visualized visualises visualisation follow immediate Immediately cardiologist Medical bike enter removed remove removing removal blood severe severely moderate moderately mild mildly view viewed viewing views due seen impair impaired impairment abnormal abnormalities satisfactorily features featuring featured abnormality grade stop stopped stopping stops workload conclusion concluded watts findings finding followup uneven unable poor image imaged imaging Leading lead department departments availability available echocardiograph electrocardiogram report reported reports reporting complications complication complicated complicate protocol predict prediction predictions predicting predicted response induce change changed changing bicycle chest achieve achieves achieved assess assessment accord accordingly according resting pain conclusions test tested following obtain obtained obtaining central centrally
reach reached reaching acquire acquired acquiring defect defective defects release Performed Performing machine accordance echocardiogram significant significance raised pattern consistent limit limited limitation limitations surgerical repair repaired repairing replacement replaced replacing departmental accurate definition region regional regions determine preserve Preserved patient patients symptom symptoms symptomatic minutes TARGETED tolerance appropriate booked error errors caution interpret interpreted evidence adjusted adequately drop estimate estimated context visual visually remains appear appeared appearance overall supin method observe observation technician detail

Figure S1. Exclusion words used in Clustering visualisation.

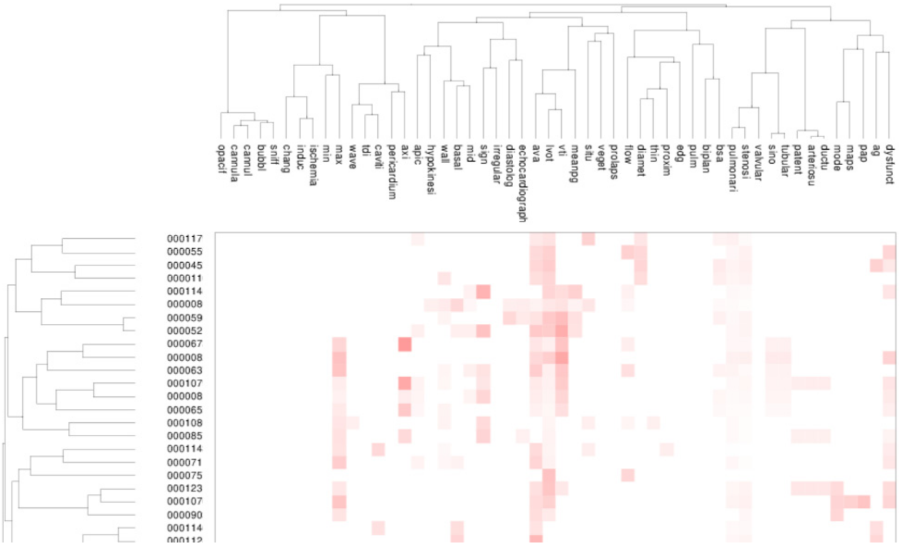

Figure S2. Automatic clustering visualisation. Rows show clustering by document number, while columns show clustering by variables that are similar across documents.

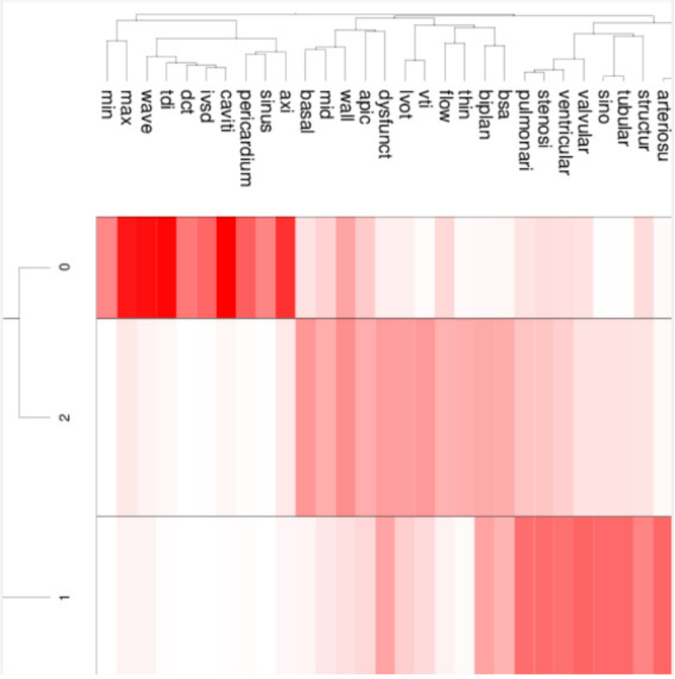

Figure S3. Clustering visualisation using cluster size of 3. Rows show three clusters forming across the documents

Table S1. Examples of JAPE rules used for matching Echo outcome measures and their corresponding values.[1]

Echo test	Regular expression for Echo variable	Regular expression for value
**AV VTI**	Rule: AorticVTIValueRule<br>Priority:1<br>(<br>(<br>({Token.string !=~ "(?i)LEFT", Token.string !=~ "(?i)RIGHT"} {Token.string ==~ "(?i)AV"} | {Token.string ==~ "(?i)AO"} | ({Token.string ==~ "(?i)AORTIC"} {Token.string ==~ "(?i)VALVE"}))<br>({Token.string !=~ "(?i)AORTA", !Lookup.majorType == aortic_TricuspidValve_Exclusion, Token.position != "startpunct", Token.string !=~ "(?i)AVA[I]?", Token.string !=~ "(?i)AR", Token.string !=~ "(?i)LAVV", Token.string !=~ "(?i)RAVV", Token.string !=~ "(?i)LEFT", Token.string !=~ "(?i)LV", Token.string !=~ "(?i)RV", Token.string !=~ "(?i)RIGHT", Token.string !=~ "(?i)LVOT", Token.string !=~ "(?i)VENTRICLE"}|{SpaceToken.string!=LVOT} | {Token.string ==~ "(?i)DESCENDING"} {Token.string ==~ "(?i)AORTA"})*<br><br>):context<br>)<br><br>(<br>(<br>{Token.string ==~ "(?i)VTI"}<br><br>({Token.string == "="} | {Token.string == "["} | {Token.string == ":"} | {Token.string ==~ "(?i)IS"} | {Token.string == " "})?<br>):context	(({Token.kind == "number"})?<br>({Token.string == "."})?<br>{Token.kind == "number"}):varValue<br><br>(<br>(({Token.string ==~ "(?i)CM"}):unit)<br>)

<table>
<tr><th>Echo test</th><th>Regular expression for Echo variable</th><th>Regular expression for value</th></tr>
<tr><td>Aortic regurgitation (AR)</td><td>Macro: aorticRegurgSeverityTerm
(
({Token.string ==~ "(?i)MINIMAL"} | {Token.string ==~ "(?i)MINIMUM"} | {Token.string ==~ "(?i)TRIVIAL"} | {Token.string ==~ "(?i)VERY"} {Token.string ==~ "(?i)MILD"} | {Token.string ==~ "(?i)TRACE"}):trivial |
({Token.string ==~ "(?i)MILD"} | {Token.string ==~ "(?i)TRIVIAL-MILD"}):mild |
({Token.string ==~ "(?i)MODERATE"} | {Token.string ==~ "(?i)MILD-MODERATE"} | {Token.string ==~ "(?i)MOD"}):moderate |
({Token.string ==~ "(?i)HEAVY"} | {Token.string ==~ "(?i)MARKED"} | {Token.string ==~ "(?i)PROMINENT"} | {Token.string ==~ "(?i)SEVERE"} | {Token.string ==~ "(?i)MODERATE-SEVERE"} | {Token.string ==~ "(?i)MODERATE"}
{Token.string ==~ "(?i)TO"} {Token.string ==~ "(?i)SEVERE"}):severe
)

Macro: aorticRegurgitationTerm
(
{Token.string ==~ "(?i)REGURGITATION"} | {Token.string ==~ "(?i)AR"} | {Token.string ==~ "(?i)AORTIC"} {Token.string ==~ "(?i)REGURGITATION"}
)</td><td>if (matchedtrivial != null) {

newFeatures.put("varValue", "1");
} else if (matchedmild != null) {

newFeatures.put("varValue", "2");
} else if (matchedmoderate != null)
{

newFeatures.put("varValue", "3");
} else if (matchedsevere != null) {

newFeatures.put("varValue", "4");
} else {

newFeatures.put("varValue", "0");
}

newFeatures.put("unit", "scale 0 (no regurgitation) to 4 (Severe regurgitation)");
newFeatures.put("varType","AR level");
newFeatures.put("context", stringFor(doc, matchedcontext));
newFeatures.put("rule", "AR level normal");</td></tr>
</table>

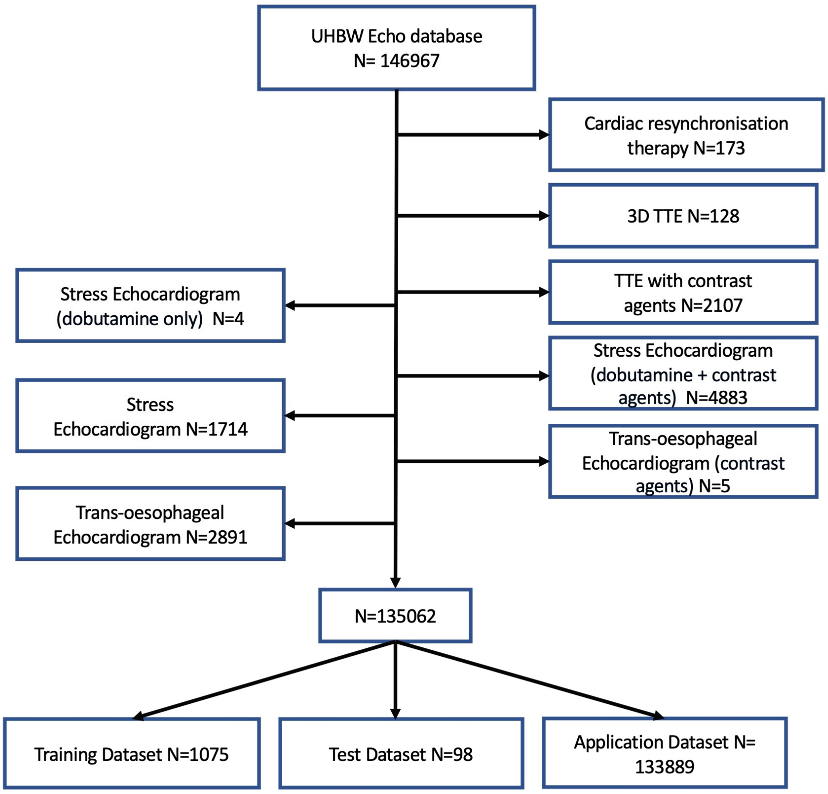

Figure S4. Consort diagram showing flow of Echocardiogram reports through the study.

References

1 Khalifa A, Meystre S. Adapting existing natural language processing resources for cardiovascular risk factors identification in clinical notes. *Journal of Biomedical Informatics* 2015;**58**:S128–32. doi:10.1016/j.jbi.2015.08.002
